# *Chroogomphus rutilus* Regulates Bone Metabolism to Prevent Periodontal Bone Loss during Orthodontic Tooth Movement in Osteoporotic Rats

**DOI:** 10.3390/nu15234906

**Published:** 2023-11-24

**Authors:** Ying Zhou, Yanfeng Zhu, Xinghui Jin, Yongfeng Zhang, Jiyu Song, Zhina Wu, Yutong Li, Jingzheng Yi, Di Wang, Min Hu

**Affiliations:** 1 Department of Orthodontics, Hospital of Stomatology, Jilin University, Changchun 130021, China; zhouying21@mails.jlu.edu.cn (Y.Z.); songjy20@mails.jlu.edu.cn (J.S.); znwu22@mails.jlu.edu.cn (Z.W.); liyutong@jlu.edu.cn (Y.L.); 2 Jilin Provincial Key Laboratory of Tooth Development and Bone Remodeling, Changchun 130021, China; 3 School of Life Sciences, Jilin University, Changchun 130012, China; zhuyf21@mails.jlu.edu.cn (Y.Z.); jinxh21@mails.jlu.edu.cn (X.J.); 4 Engineering Research Center of Chinese Ministry of Education for Edible and Medicinal Fungi, Jilin Agricultural University, Changchun 130118, China; zhangyongfeng@jlau.edu.cn; 5 Western Dental Kids, Fresno, CA 93726, USA; yijingzheng1994@gmail.com

**Keywords:** fungus, *Chroogomphus rutilus*, orthodontic tooth movement, osteoporosis, inflammation, Wnt/β-catenin, dentistry, orthodontics

## Abstract

Osteoporosis (OP) leads to the acceleration of tooth movement and aggravation of periodontal bone loss during orthodontic treatment. *Chroogomphus rutilus* (CR) is abundant in nutrients and demonstrates remarkable antioxidant and anti-inflammatory properties. In the present study, the components of CR, including 35.00% total sugar, 0.69% reducing sugar, 14.40% crude protein, 7.30% total ash, 6.10% crude fat, 0.51% total flavonoids, 1.94% total triterpenoids, 0.32% total sterol, 1.30% total saponins, 1.69% total alkaloids, and 1.02% total phenol, were first systematically examined, followed by an investigation into its regulatory effects on bone metabolism in order to mitigate bone loss during orthodontic tooth movement in osteoporotic rats. The results of the imaging tests revealed that CR treatment reduced periodontal bone loss and normalized tooth movement in the OP. In conjunction with analyses of intestinal flora and metabolomics, CR enhances the prevalence of anti-inflammatory genera while reducing the production of inflammatory metabolites. Meanwhile, CR reduced the levels of periodontal inflammatory factors, including TNF-α, IL-1β, and IL-6, by activating Wnt/β-catenin signaling, and promoted periodontal bone formation. These findings imply that CR is a potent supplementary therapy for controlling periodontal bone remodeling in patients with OP undergoing orthodontic treatment.

## 1. Introduction

Orthodontic tooth movement (OTM) is a periodontal bone tissue remodeling process mediated by mechanics in which the balance between bone formation, on the tension side, and bone resorption, on the pressure side, maintains the stability of tooth movement [1]. Unfortunately, patients with abnormal bone metabolism, such as those with osteoporosis (OP), show increased osteoclast formation and bone resorption as well as a common decrease in alveolar bone density [2], which aggravates tooth root resorption and excessive alveolar bone resorption, making it difficult to maintain orthodontic effects and resulting in poor long-term stability of orthodontic treatment [1,3].

Drugs, including bisphosphonates (BPs), calcitonin, and estrogen, are frequently used to treat OP [4]. Although BPs are currently the most commonly used anti-OP medications in clinical practice, their side effects, such as gastrointestinal problems and jaw osteonecrosis, have drawn clinical attention [5]. In orthodontics, tooth movement is precisely regulated by momentarily suppressing and resuming movement; however, BPs hinder such control because of their long half-life of several years in the bone [6]. Therefore, alternative therapeutic drugs that possess reduced side effects and lower prices are urgently required for patients undergoing osteoporotic orthodontics.

The gut–bone axis has been proposed as a result of mounting evidence demonstrating a strong relationship between gut microbiota and bone metabolism. An ecological imbalance in the intestinal flora can lower mucin production, which increases the production of pro-inflammatory cytokines. Intestinal permeability increases, and the integrity of the epithelial layer is compromised, consequently increasing the levels of metabolites linked to OP that encourage bone resorption [7]. Probiotics can improve the intestinal barrier, reduce aberrant bone metabolism, and hence reduce bone loss in OP, further highlighting the importance of the intestinal barrier in the development and management of OP [8].

Edible fungi are nutritious, functional, safe, and capable of alleviating OP [9,10]. *Cordyceps* can enhance osteoblast differentiation [11] and prevent the development of OP in rats [9]. Similarly, *Pleurotus ostreatus* and *Ganoderma lucidum* exhibit potent osteogenic activities [10]. *Chroogomphus rutilus* (CR) is a basidiomycete belonging to the Rivetaceae family and Agaricus orders and is widely distributed in Northeast China, Hebei, and Yunnan regions. The findings of previous studies have demonstrated that the polyphenolic extract of CR exhibits strong antioxidant and anti-inflammatory activities [12]. The ethanol extract of CR can suppress serum levels of triglycerides, low-density lipoprotein cholesterol, and total cholesterol while raising high-density lipoprotein cholesterol levels, demonstrating superior blood lipid-lowering efficacy [13]. Interestingly, abnormal lipid metabolism, oxidative stress, and inflammation are the key factors in the etiology of OP.

CR, given its anti-inflammatory properties, would exhibit regulatory effects on bone metabolism, thereby preventing bone loss during OTM in osteoporotic rats.

In this study, we aimed to examine the potential effects of CR on periodontal bone loss, inflammatory factors, and the associated signaling pathways in the context of orthodontic treatment in osteoporotic conditions, providing an experimental basis for its adjuvant application in OP treatment.

## 2. Materials and Methods

### 2.1. CR Component Detection

CR fruiting bodies were purchased from the Chinese market in Chengde, Hebei Province. The fruiting bodies were pulverized in a blender and subsequently dehydrated. Six minerals, five nucleotides, 17 amino acids, and six heavy metals were systematically analyzed in CR fruiting bodies, along with eleven general nutrients (total sugar, reducing sugar, crude protein, total ash, crude fat, total flavonoids, total triterpenoids, total phenol, total saponins, total alkaloids, and total sterol), as described in previous studies [14,15,16,17,18,19].

### 2.2. Animal Experiments and Agent Administration Protocol

All animal experimental procedures were approved by the Institutional Animal Ethics Committee of Jilin University (SY202201011). Sixty Sprague Dawley rats (male; 6–8 weeks old; 200–220 g), purchased from Liaoning Changsheng Biotechnology Co., Ltd. (SCXK [LIAO] 2020-0001; Liaoning, China), were given sterile food and water while being housed in a pathogen-free environment with a 12 h light/12 h dark cycle.

After being fed adaptively for one week, four groups of rats were randomly assigned as follows: normal control rats (CTRL) (*n* = 15) were intraperitoneally injected with normal saline and intragastrically administered double-distilled (D.D.) water daily. The vehicle-treated OP rats (*n* = 15) were intraperitoneally injected with dexamethasone (Dex; 20 mg/kg; Zhuofeng Pharmaceutical; Zhengzhou, China) and intragastrically administered D.D. water daily. The positive control rats (*n* = 15) were intraperitoneally injected with Dex (20 mg/kg), and intragastrically administered calcitriol (Cal; 40 ng/kg; Zhengda Pharmaceutical; Qingdao, China) daily. The CR-treated OP rats (*n* = 15) were intraperitoneally injected with Dex (20 mg/kg), and intragastrically administered CR (1.5 g/kg) daily.

After the 42nd day of administration, rats treated with Dex were characterized as having OP, and then, based on previous studies, orthodontic appliances were installed for all rats [1]. Briefly, rats were anesthetized with 1% pentobarbital (Xinya Pharmaceutical Co., Ltd.; Shanghai, China), and a nickel–titanium closed-coil spring appliance (Xinya Medical Technology Co., Ltd.; Zhejiang, China) was applied to establish an OTM model of the left maxillary first molar. Throughout the research, daily checks were made on the equipment and condition of the animals. Three days after the device was installed, a soft diet was offered, and pain management was applied when symptoms of discomfort appeared. Rats’ weights were monitored weekly throughout the experiment. Rats were euthanized after the 14th day of tooth movement, and the serum, cecal contents, and main organs, such as the heart, liver, spleen, kidney, femur, tibia, and maxillary bone, were collected for pathological analysis (Figure S1A).

### 2.3. X-ray and Micro-Computed Tomography (Micro-CT) Analysis

After being fixed in 4% paraformaldehyde (PFA; BL539A; Biosharp; Shanghai, China) for three days, the femur and tibia were examined utilizing X-ray imaging (UltraFocus 10 × 15; Faxitron; Tucson, AZ, USA). Alveolar bones were scanned (voltage, 70 kV; current, 200 μA; integration time, 300 ms; and voxel size, 20 μm) using a micro-CT 50 system (μCT-50; Scanco Medical; Bruttisellen, Switzerland) in the standard resolution mode. The images were utilized for the reconstruction of three-dimensional imagery (Skyscan1172; Bruker; Kontich, Belgium). The region of interest (ROI) included the interradicular area of the left maxillary first molar (200 × 200 × 200 μm). The ROI’s structural parameters were calculated using the same methodology as in the previous study [1,20].

### 2.4. Intestinal Microflora Analysis

The cecal contents were aseptically collected in sterile EP tubes (615601; NEST Biotechnology Co., Ltd.; Wuxi, China) at the conclusion of the drug treatment (*n* = 3/group). The bacterial 16s rRNA gene’s V3 to V4 region was amplified through polymerase chain reaction (PCR) after the microbial DNA was extracted [21]. Paired-end 2 × 250 bp fragments were sequenced on the Illumina MiSeq platform using the MiSeq Reagent Kit v3 (Personal Biotechnology; Shanghai, China), as described in our previous study [21]. The operational taxonomic units (OTUs), which were identified with 97% similarity, were clustered using the Vsearch method. Based on the Greengenes database, the classify-sklearn algorithm, QIIME2 (2019.4), was used for species annotation. The evaluation indicators of alpha diversity were used as post-hoc tests to verify the significance of the differences. To evaluate beta diversity, principal coordinates analysis (PCoA), which concentrates on variations between sample groups discovered using the Jaccard distance matrix, was utilized. The metabolic and functional pathways connected to the altered gut flora in CR-treated OP rats were discovered by examining the metabolic pathways across all domains of life using the MetaCyc database.

### 2.5. Non-Targeted Metabolomics Analysis

Serum samples were subjected to analysis using ultra-high-performance liquid chromatography–tandem mass spectrometry (UPLC-MS/MS)-based non-targeted metabolomics by Personal Biotechnology Co., Ltd. (Shanghai, China), as previously described [22]. To analyze the variance in metabolites, orthogonal partial least squares discriminant analysis (OPLS-DA) and Pareto-scaled principal component analysis (PCA) were utilized. To identify the potentially relevant metabolic indicators, the significance of the expression levels of metabolites was assessed using a variable importance in projection (VIP) value > 1 and a *p*-value < 0.05. The linked metabolic and signal transduction pathways of the significantly differentially expressed metabolites in CR-treated OP rats were investigated utilizing the Kyoto Encyclopedia of Genes and Genomes (KEGG).

### 2.6. Histopathological Analysis and Immunohistochemical Examination

The organ tissue samples (heart, liver, spleen, and kidneys) selected from rats in all groups were fixed in 4% PFA for 24 h (the alveolar bones were decalcified with ethylene diamine tetraacetic acid (G1105; Servicebio; Wuhan, China) for 6 weeks, dehydrated with ethanol, embedded in paraffin, cut into 5 μm sections, and treated with hematoxylin and eosin (H&E) staining (B1000; Baiqiandu Biotechnology; Wuhan, China), as previously described [22].

For immunohistochemical (IHC) staining [22], alveolar bones were incubated alternately with antigen retrieval solution and 3% H_2_O_2_ for 25 min, washed with phosphate-buffered saline, incubated for 24 h at 4 °C with the primary antibody (Table S1), and incubated with HRP-labeled secondary antibodies at room temperature for 30 min, then counterstained with hematoxylin.

### 2.7. Statistical Analysis

All values are displayed as mean ± SEM. The Shapiro–Wilk test was used for the normality test. The biochemical indices were compared among different groups using one-way analysis of variance (ANOVA), followed by a post-hoc multiple comparisons (Tukey) test conducted with DSS 25.0 software (IBM, Armonk, NY, USA). The threshold for statistical significance was *p* < 0.05.

## 3. Results

### 3.1. Main Composition of CR

CR contained 35.00% total sugar, 0.69% reducing sugar, 14.40% crude protein, 7.30% total ash, 6.10% crude fat, 0.51% total flavonoids, 1.94% total triterpenoids, 1.02% total phenols, 0.30% total saponins, 1.69% total alkaloids, and 0.32% total sterols. Glutamic acid and uracil nucleotides comprised the highest content of the detected amino acids and nucleotides, respectively. The presence of six minerals, namely Ca, Fe, Zn, K, Na, and Mn, was detected, while the concentration levels of heavy metals such as Pb, As, Hg, Cd, Cu, and Cr were found to be low in the CR (Table 1).

### 3.2. CR Promoted Bone Remolding in Osteoporotic Alveolar Bone during OTM

Sustained Dex injection significantly lowered the body weight (*p* < 0.001) while increasing the indices of the heart (*p* < 0.001), liver (*p* < 0.001), and kidney (*p* < 0.001) in comparison to those of CTRL rats (Table S2). Other than a substantial reduction in the liver index (*p* < 0.001), CR treatment had no discernible impact on body weight or indices of other organs in OP rats (Table S2). The H&E staining revealed no notable pathological alterations in the heart, liver, or kidneys (Figure S1B,C,E). The spleen displayed a disorganized architecture with no discernible separation between the red and white pulp in vehicle-treated OP rats, whereas intervention with CR strongly reversed these changes (Figure S1D).

**Figure 1:**
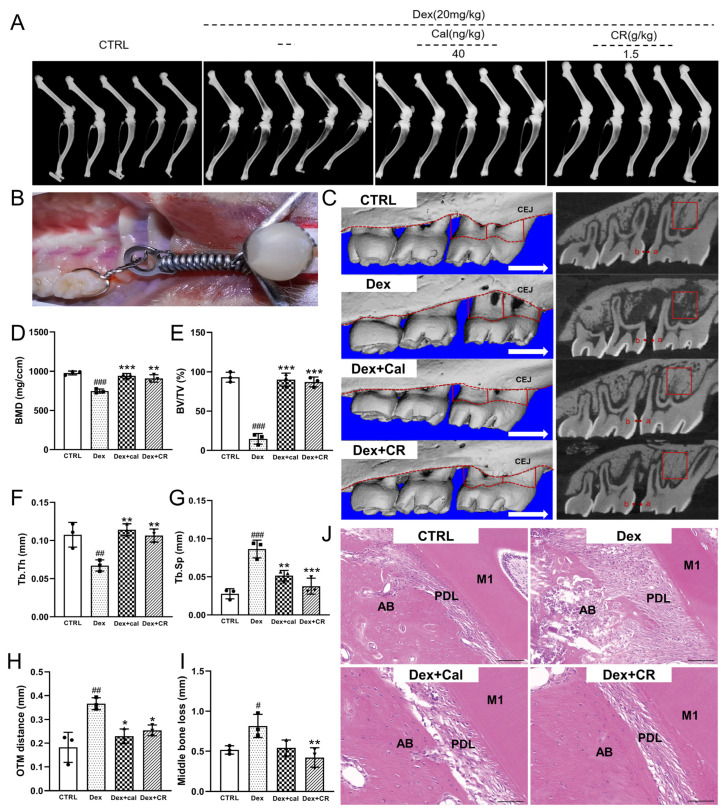
CR promoted bone remodeling in osteoporotic alveolar bone during OTM. (**A**) X-rays of femur and tibia in experimental rats (*n* = 5/groups). (**B**) Intraoral schematic of the OTM model. (**C**) CT scan of the left maxilla (*n* = 3/groups): 3D reconstructions of maxillary samples are shown on the left. The red dotted lines indicate the cementoenamel junction (CEJ; upper) and the alveolar crest (lower). Red solid line indicates the mesial, middle, and distal alveolar bone loss of the first molar. The white arrow points to the direction of tooth movement. The right side is the sagittal view of the alveolar bone; the a and b points represent the OTM distance, and the red box represents ROI of the alveolar bone. Quantitative analyses of (**D**) BMD, (**E**) BV/TV, (**F**) Tb.Th, (**G**) Tb.Sp, (**H**) OTM distance, and (**I**) middle bone loss among groups (*n* = 3/group). (**J**) H&E staining of alveolar bone (*n* = 3/group, 200×, scale bar: 100 µm). M1: first molar; PDL: periodontal ligament; AB: alveolar bone. Data are expressed as mean ± SEM, and were analyzed via one-way ANOVA. ^#^ *p* < 0.05, ^##^ *p* < 0.01, ^###^ *p* < 0.001 *versus* CTRL rats; * *p* < 0.05, ** *p* < 0.01, *** *p* < 0.001 *versus* vehicle-treated OP rats. (CR: *Chroogomphus rutilus*; OTM: orthodontic tooth movement; CT: computed tomography; ROI: the region of interest; BMD: bone mineral density; BV/TV: bone volume/total volume; Tb.Th: trabecular thickness; Tb.Sp: trabecular separation/spacing; H&E: hematoxylin and eosin).

According to X-ray analysis, in vehicle-treated OP rats, diminished radiation absorption in the femur and tibia, as well as thinner cortical bone, were noted compared with those in the control rats, both of which were remarkably rectified following CR intervention (Figure 1A).

To investigate the effect of CR on alveolar bone remodeling in osteoporotic rats undergoing OTM, alveolar bones were collected two weeks after tooth movement, and micro-CT was used to analyze the alveolar bone mass and microstructural destruction (Figure 1B,C). Compared to vehicle-treated OP rats, CR significantly reduced alveolar bone resorption in OP rats (Figure 1C,I). Based on standard 3D microstructural analysis, decreased levels of bone mineral density (BMD) (*p* < 0.001) (Figure 1D), bone volume/total volume (BV/TV) (*p* < 0.001) (Figure 1E), and trabecular thickness (Tb.Th) (*p* < 0.01) (Figure 1F), and enhanced levels of trabecular separation/spacing (Tb.Sp) (*p* < 0.001) (Figure 1G) were noted in vehicle-treated OP rats compared with those of CTRL rats. Additionally, the upper first molars of vehicle-treated OP rats moved greater distances (*p* < 0.01) (Figure 1H) than those of CTRL rats, indicating faster tooth movement related to abnormal bone metabolism. All the above pathological changes in bone parameters were strongly reversed by CR (BMD, *p* < 0.01, Figure 1D; BV/TV, *p* < 0.001, Figure 1E; Tb.Th, *p* < 0.01, Figure 1F; Tb.Sp, *p* < 0.001, Figure 1G; middle bone loss, *p* < 0.01, Figure 1I); meanwhile, the speed of tooth movement was slowed by CR (*p* < 0.05) (Figure 1H). However, mesial and distal bone loss did not change significantly (Figure S2). According to the H&E staining, severe alveolar bone resorption, irregular arrangement of bone trabecula, and enlarged bone marrow cavities in vehicle-treated OP rats were reversed by CR (Figure 1J). CR helped reduce alveolar bone resorption during OTM in OP and maintained a stable state of alveolar bone mass.

### 3.3. CR Preserved the Balance of Intestinal Flora in OP Rats

**Figure 2:**
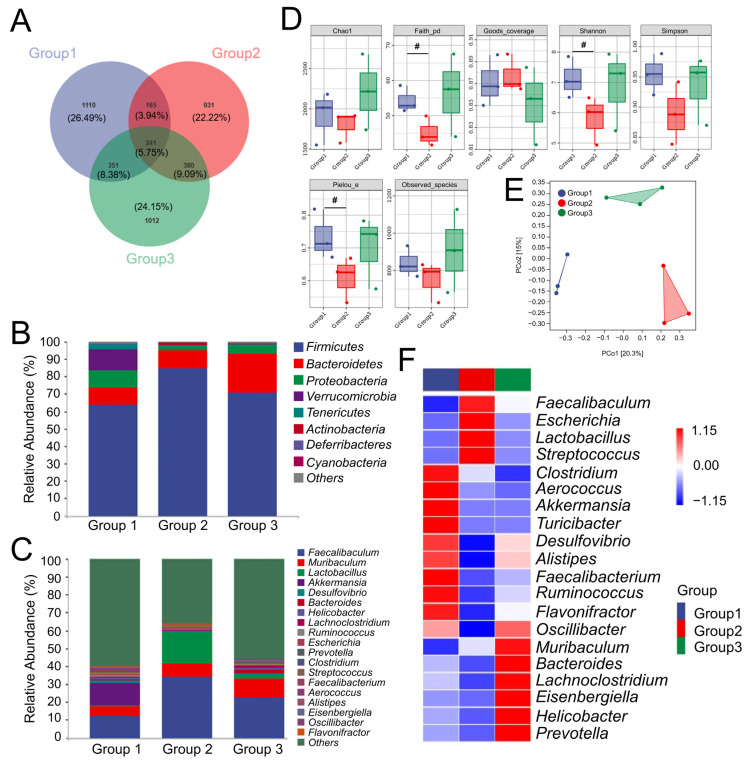
CR preserved the balance of intestinal flora in osteoporosis (OP) rats (*n* = 3/group). (**A**) Venn diagram. The top 10 phylum (**B**) and top 20 genera (**C**) in terms of intestinal abundance. (**D**) α-diversity analysis. (**E**) PCoA plots represented as β-diversity relying on the Jaccard distance algorithm. (**F**) Heatmap of the top 20 genera in abundance. Group 1: CTRL rats; Group 2: vehicle-treated OP rats; Group 3: CR-treated OP rats. ^#^ *p* < 0.05 *versus* CTRL rats. (CR: *Chroogomphus rutilus*).

According to the Venn diagram, 4190 OTUs were found across detected groups based on a 97% similarity threshold. In total, 1110 (26.49%), 931 (22.22%), and 1012 (24.15%) specific OTUs were found in the CTRL group, vehicle-treated OP group, and CR-treated OP groups, respectively (Figure 2A). At the phylum level, the microbial community composition of the vehicle-treated OP group rats mainly consisted of *Firmicutes* (84.58%), *Bacteroidetes* (10.43%), and *Proteobacteria* (2.64%) (Figure 2B). In contrast, CR administration increased the proliferation of *Bacteroidetes* to 22.48% and decreased the relative abundance of *Firmicutes* to 70.56% (Figure 2B). Further analysis was performed on the top 20 abundance-varied bacterial genera in the three groups. Compared with the vehicle-treated OP group rats, CR enhanced the abundance of *Muribaculum*, *Desulfovibrio*, *Bacteroides*, *Helicobacter*, *Lachnoclostridium*, *Ruminococcus*, *Prevotella*, *Faecalibacterium*, *Alistipes*, *Eisenbergiella*, *Oscillibacter*, and *Flavonifractor* and reduced the abundance of *Streptococcus*, *Aerococcus*, *Clostridium*, *Escherichia*, *Lactobacillus*, and *Faecalibaculum* (Figure 2C,F; Table S3). Compared with the CTRL group, α-diversity, especially Faith’s phylogenetic diversity and Shannon and Pielou’s evenness index values, were significantly decreased in the vehicle-treated OP group (*p* < 0.05); however, CR failed to influence these parameters (Figure 2D). Significant variations in the composition of the gut microbial community among groups were shown by PCoA plots relying on the Jaccard distance algorithm, which is represented as β-diversity (Figure 2E). Based on the MetaCyc database, within 7 primary functional pathways, 55 secondary functional pathways were analyzed; fatty acid and lipid biosynthesis (abundance: 13,388.04), fatty acid and lipid degradation (33.62), amino acid biosynthesis (41,396.53), amino acid degradation (398.23), and TCA cycle (2130.98) were related to bone metabolism (Figure S3).

### 3.4. CR Modified the Metabolite Levels in Serum of OP Rats

**Figure 3:**
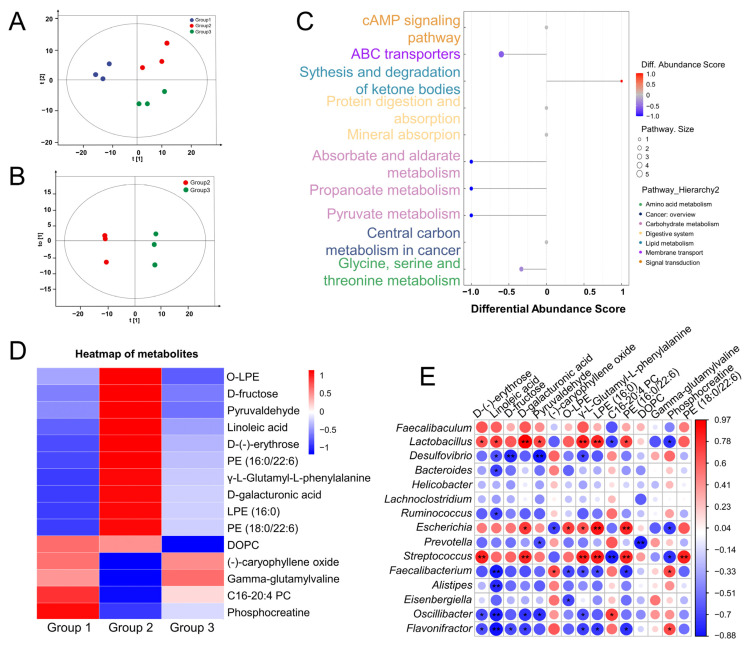
CR modified the metabolite levels in serum of osteoporosis (OP) rats (*n* = 3/group). (**A**) PCA score plots among three groups. (**B**) OPLS-DA plots between Group 2 and Group 3. (**C**) KEGG pathway analysis predicts alterations in metabolic pathways between vehicle-treated OP rats and CR-treated OP rats. (**D**) Heatmap of significantly changed serum metabolites. (**E**) Joint analysis of serum metabolites and gut microbiota. Group 1: CTRL rats; Group 2: vehicle-treated OP rats; Group 3: CR-treated OP rats. * *p* < 0.05, ** *p* < 0.01 between serum metabolites and intestinal flora. (CR: *Chroogomphus rutilus*; PCA: principal component analysis; OPLS-DA: orthogonal partial least squares discriminant analysis; KEGG: Kyoto Encyclopedia of Genes and Genomes).

In total, 917 serum metabolites, including organic acids and derivatives (24.76%), lipids and lipid-like molecules (23.45%), organoheterocyclic compounds (13.96%), and others, were found in the experimental groups (Figure S4A). Metabolomics data differentiations between CTRL-, vehicle-, and CR-treated OP rats were seen in PCA score plots (Figure 3A). Between CTRL and vehicle-treated OP groups, remarkable differences in OPLS-DA score plots were noted, suggesting severe metabolic abnormalities in OP rats (Figure S4B). Similarly, distinct separation between the vehicle-treated and CR-treated OP groups could also be noted in the OPLS-DA plots (Figure 3B). The permutation test was employed in the modeling process to mitigate overfitting of the supervised model and ensure its validity. Figure S5 shows the permutation test plots of the OPLS-DA model between experimental groups. The gradual decrease in permutation retention was accompanied by a corresponding decline in both R2 and Q2 values of the random model, indicating the absence of any overfitting phenomenon in the original model. A total of 143 metabolites were markedly different between vehicle-treated OP and CTRL groups (based on VIP > 1.0, *p* < 0.05) (Figure S4C), and 48 metabolites were significantly different between the vehicle-treated and CR-treated OP groups (Figure S4D). According to the KEGG analysis, CR altered five metabolic pathways, which included glycine, serine, and threonine metabolism; ABC transporters; synthesis and degradation of ketone bodies; absorbate and aldarate metabolism; propanoate metabolism; and pyruvate metabolism (Figure 3C). Heatmap analysis revealed that CR dramatically increased the relative amounts of (-)-caryophyllene oxide, gamma-glutamylvaline, C16-20:4 PC, and phosphocreatine, while reducing the relative contents of D-(-)-erythrose, linoleic acid, D-fructose, D-galacturonic acid, pyruvaldehyde, DOPC, LPE (16:0), PE (16:0/22:6), PE (18:0/22:6), gamma-L-glutamyl-L-phenylalanine, and O-LPE in OP rats (Figure 3D; Table S4). The association between the bacterial genera and various metabolites altered by CR therapy in OP rats was further investigated using Spearman’s correlation coefficient. *Alistipes* and *Oscillibacter* were negatively correlated with linoleic acid content. *Escherichia* and *Streptococcus* were positively corrected with LPE (16:0) and PE (16:0/22:6) (Figure 3E).

### 3.5. CR Regulated Wnt/β-Catenin Signaling to Promote Periodontal Bone Formation

**Figure 4:**
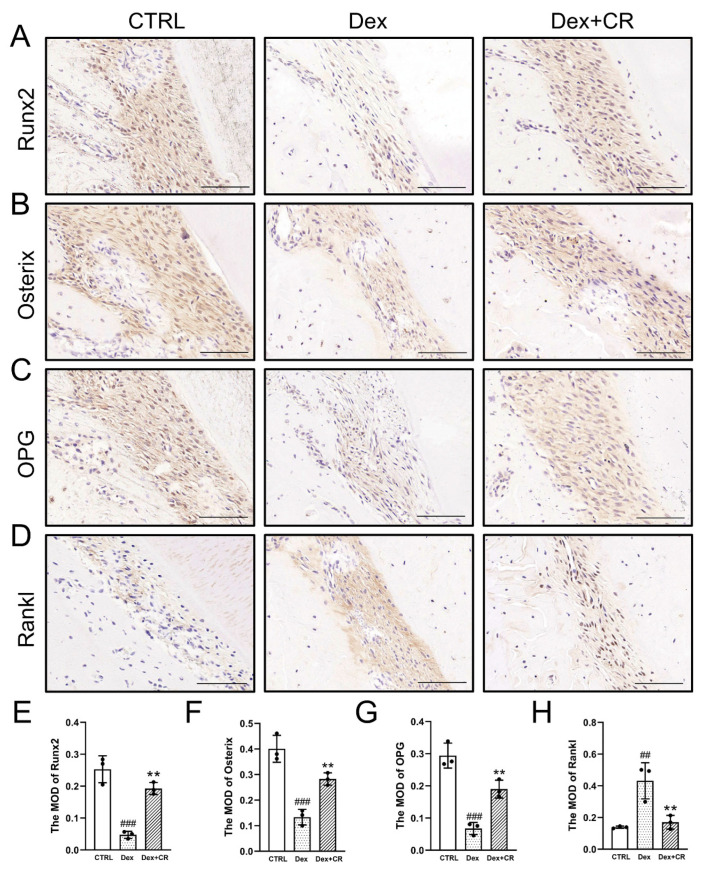
CR promoted the expression of periodontal osteogenic differentiation marker factors during OTM in OP rats. IHC staining of (**A**) Runx2, (**B**) osterix, (**C**) OPG, and (**D**) RANKL in periodontal tissue on the tension side of OTM (*n* = 3/group, 200×, scale bar: 100 µm). Quantification of MOD of (**E**) Runx2, (**F**) Osterix, (**G**) OPG, and (**H**) RANKL in the tension area (*n* = 3/group). Data are expressed as the mean ± SEM and were analyzed via one-way ANOVA. ^##^ *p* < 0.01 and ^###^ *p* < 0.001 *versus* CTRL rats; ** *p* < 0.01 *versus* vehicle-treated OP rats. (CR: *Chroogomphus rutilus*; IHC: immunohistochemical; Runx2: runt-related transcription factor 2; OPG: osteoprotegerin; RANKL: receptor activator of NF-κB ligand; MOD: mean optical density).

**Figure 5:**
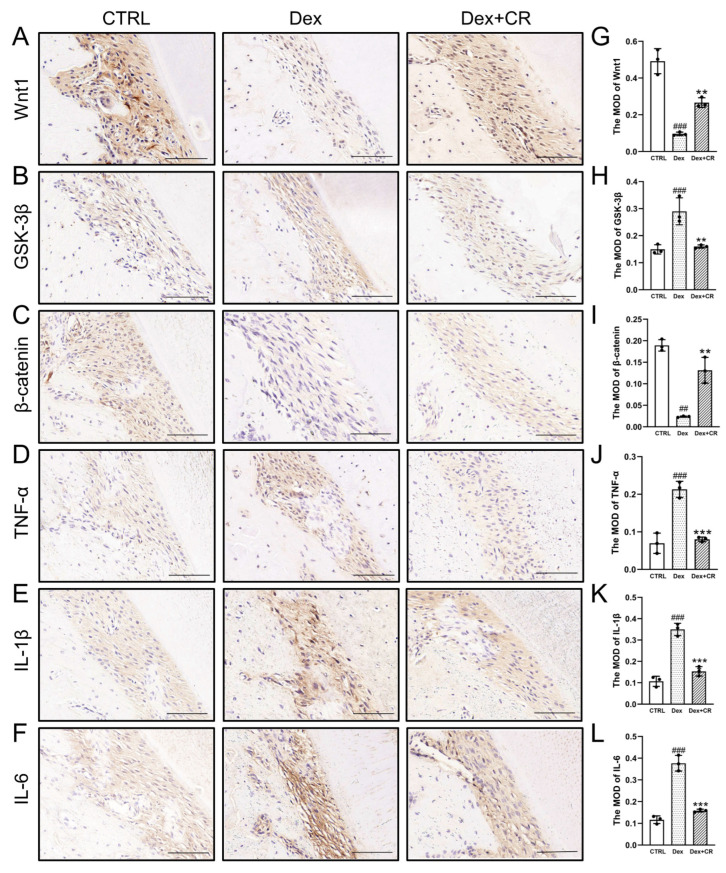
CR regulated Wnt/β-catenin signaling and reduced periodontal inflammation during OTM in OP rats. IHC staining of (**A**) Wnt1, (**B**) GSK-3β, (**C**) β-catenin, (**D**) TNF-α, (**E**) IL-1β, and (**F**) IL-6 in periodontal tissue on the tension side of OTM (n = 3/group, 200×, scale bar: 100 µm). Quantification of MOD of (**G**) Wnt1, (**H**) GSK-3β, (**I**) β-catenin, (**J**) TNF-α, (**K**) IL-1β, and (**L**) IL-6 in the tension area (*n* = 3/group). Data are expressed as the mean ± SEM and were analyzed via one-way ANOVA. ^##^ *p* < 0.01 and ^###^ *p* < 0.001 *versus* CTRL rats; ** *p* < 0.01 and *** *p* < 0.001 *versus* vehicle-treated OP rats. (CR: *Chroogomphus rutilus*; OTM: orthodontic tooth movement; IHC: immunohistochemical; GSK-3β: glycogen synthase kinase-3β; TNF-α: tumor necrosis factor-alpha; IL: interleukin; MOD: mean optical density).

An increased level of periodontal inflammation is closely related to periodontal bone loss in the OP state, and the Wnt/β-catenin signaling pathway serves as the pivotal connection between inflammation and bone resorption [23]. CR significantly increased the expression levels of osteogenic marker factors, including runt-related transcription factor 2 (Runx2) (*p* < 0.01), Osterix (*p* < 0.01), and osteoprotegerin (OPG) (*p* < 0.01), and down-regulated the expression of the receptor activator of the NF-κB ligand (RANKL) (*p* < 0.01) (Figure 4), indicating that CR can restore the osteogenic differentiation ability of periodontal tissue in OP rats. CR strongly enhanced the levels of Wnt1 (*p* < 0.01) and β-catenin (*p* < 0.01) and suppressed the levels of glycogen synthase kinase-3β (GSK-3β) (*p* < 0.01) (Figure 5A–C,G–I) in the periodontal tissue on the tension side of OP rats. Moreover, TNF-α (*p* < 0.001), IL-1β (*p* < 0.001), and IL-6 (*p* < 0.001) were significantly downregulated by CR (Figure 5D–F,J–L). These data suggest that CR suppressed periodontal inflammation in OP rats and promoted periodontal bone formation partially through the Wnt/β-catenin pathway.

## 4. Discussion

As a functional fungus, CR is rich in nutrients, among which the polysaccharide content (35.00%) is the highest. Notably, the polysaccharides derived from fungi demonstrate remarkable anti-OP activity. For example, *Ganoderma lucidum* polysaccharides can reduce alveolar bone loss and periodontal inflammation [24]. *Poria cocos* polysaccharides inhibit osteoclastogenesis and prevent diseases related to excessive bone loss [25]. In this study, we first report the promotion of CR on bone remodeling in osteoporotic rats during OTM.

The alveolar bone, which is the most metabolically and remodelingly active part of the skeletal system, is essential for the stability of orthodontic therapy. Gut flora participates in generalized metabolic processes and is vital for the regulation of alveolar bone remodeling [26]. The administration of CR resulted in an augmentation of beneficial bacteria, such as *Bacteroides*, *Alistipes*, and *Oscillibacter*. As a dominant genus, *Bacteroides* maintains the stability of a healthy gut ecosystem, and its decrease in abundance may predispose individuals to inflammatory diseases [27]. *Alistipes* and *Oscillibacter* preserve the permeability and integrity of the intestinal barrier and can reduce serum endotoxin levels associated with pathological bone resorption [28,29]. Moreover, CR decreased the levels of *Faecalibaculum*, resulting in high expression levels of TNF-α [30], while the decreased abundance of *Faecalibaculum* improved intestinal barrier integrity, metabolic disturbance, and remodeling composition of the gut microbiota [31]. *Firmicutes*/*Bacteroidetes*, previously reported as indicators of ecological imbalance [32], were also reversed by CR in OP mice. Accordingly, adjusting the intestinal flora may be a potential target for CR to reduce periodontal bone loss during OTM.

CR can influence the process of OP by preserving healthy lipid metabolism. In patients with OP, hyper-levels of linoleic acid, glycine, serine, and threonine in the plasma were noted [33], which were all suppressed by CR in OP rats. Phosphocreatine, which exists in muscle and other excitatory tissues, temporarily stores high-energy phosphate and promotes osteogenesis and mineralization [34], which was suppressed in CR-treated OP rats. Inflammation has a major impact on bone turnover and is an important component in the pathogenesis of OP; the local inflammatory reactions during OTM increase the susceptibility of periodontal tissue destruction and alveolar bone resorption in patients undergoing OP [3]. Glycerophospholipids are considered potential inflammatory mediators, which can promote inflammatory responses by increasing the levels of IL-1β and leukotrienes [35]. Moreover, glycerophospholipids are involved in regulating bone metabolism, which is closely related to an imbalance between bone resorption and formation [36]. It is worth noting that linoleic acid exerts an inhibitory effect on the Wnt pathway by downregulating β-catenin accumulation in the nucleus [37].

The activated Wnt signaling can inhibit the activity of GSK-3β and reduce the phosphorylation level of β-catenin; therefore, β-catenin accumulates stablely in cells and accurately enters the nucleus, where it regulates the transcription or expression of target genes such as Runx2 [38]. Up-regulation of Runx2 can accelerate osteogenic differentiation, promote the maturation of new osteoblasts, and stabilize alveolar bone and periodontal ligament remodeling [39]. As the downstream of Runx2, Osterix affects the maturation of osteoblasts [40]. CR strongly enhanced the expression of Runx2 and Osterix, suggesting that it improved osteogenic differentiation via Wnt signaling.

Wnt signaling is a key link between inflammation and bone destruction [23]. When the periodontal ligament is attacked by inflammatory factors, the balance of bone metabolism is disrupted, and alveolar bone resorption occurs. CR significantly decreased the levels of pro-inflammatory cytokines IL-1β, IL-6, and TNF-α in the periodontal tissues of OP rats. IL-6 can reduce the expression of Runx2, which is related to osteogenic differentiation, thereby reducing the bone mineralization ability of osteoblasts and inhibiting osteogenic differentiation [41]. The secretion of matrix metalloproteins can be enhanced by TNF-α, leading to the degradation of periodontal tissue [42]. Elevated TNF-α levels can cause inflammation, downregulate Runx2 and Osterix expression, and prevent osteogenic differentiation [43]. Additionally, TNF-α and IL-1β can increase the expression of RANKL and decrease the expression of OPG on the surface of osteoblasts [44]. The combination of RANKL and receptor activator of nuclear factor κB promotes the activation and differentiation of osteoclasts and inhibits the apoptosis of mature osteoclasts. Meanwhile, low levels of OPG, a competitive inhibitor of RANKL, lead to an increase in bone resorption [45]. Accordingly, CR suppressed the periodontal inflammation and inhibited periodontal bone loss during OTM. Additionally, some recently introduced compounds have been demonstrated to have a significant influence on the oral environment [46]. The use of lysates [47] and postbiotics [48] can modify clinical and microbiological parameters in periodontal patients, so these products should also be considered in future clinical trials as adjuvants in combination with CR.

This study had some limitations. Firstly, we failed to analyze the active ingredients of CR with anti-OP activity. As mentioned, polysaccharides are the predominant nutrient in CR, suggesting a potential correlation between the anti-inflammatory activity of CR and its high polysaccharide content. The structural attributes of polysaccharides generated from natural sources are significantly correlated with their biological activity [49]. A galactoglucan of *Laetiporus sulphureus* inhibited secretion of inflammatory factor in LPS-induced RAW 264.7 macrophages with promising anti-inflammatory activity [50]. Likewise, both *Pellinus* polysaccharide, primarily composed of lactose, and *Poria cocos* polysaccharide demonstrate efficacy against inflammatory disorders [51,52]. Our future research will be focused on the activities of purified polysaccharides from CR. Furthermore, considering the preclinical studies conducted on animal models, this report may not fully encompass the intricacies of human physiology and response to treatment. Hence, it is imperative to meticulously design clinical experiments in future research endeavors to assess the potential advantages of CR for human subjects.

## 5. Conclusions

In summary, CR, which is rich in components, can restore periodontal bone homeostasis in OP rats by improving bone metabolism, thereby preventing periodontal bone loss during orthodontic treatment. This effect of CR may reduce the possibility of root resorption and alveolar bone overresorption in orthodontic clinical treatment and can serve as a promising supplementary therapy to offer patients with OP safe orthodontic treatment with better and more manageable outcomes.

## Figures and Tables

**Table 1:** The main composition of *Chroogomphus rutilus* (CR).

	Compounds	Contents (%)	Compounds	Contents (%)
General nutrients	Total sugar	35.00	Total triterpenoids	1.94
	Reducing sugar	0.69	Total phenol	1.02
	Crude protein	14.40	Total saponins	0.30
	Total ash	7.30	Total alkaloids	1.69
	Crude fat	6.10	Total sterol	0.32
	Total flavonoids	0.51		
Amino acids	Aspartic acid (Asp)	0.83	Threonine (Thr)	0.44
	Serine (Ser)	0.48	Glutamic acid (Glu)	1.36
	Glycine (Gly)	0.40	Alanine (Ala)	0.52
	Cystine (Gys)	0.041	Valine (Val)	0.45
	Methionine (Met)	0.14	Isoleucine (IIe)	0.33
	Leucine (Leu)	0.69	Tyrosine (Tyr)	0.23
	Phenylalanine (Phe)	0.38	Lysine (Lys)	0.48
	Histidine (His)	0.23	Arginine (Arg)	0.52
	Proline (Pro)	0.41		
	Compounds	Contents (mg/kg)	Compounds	Contents (mg/kg)
Minerals	Calcium (Ca)	237	Iron (Fe)	328
	Zinc (Zn)	29.9	Potassium (K)	2.66 × 10^4^
	Sodium (Na)	25.7	Manganese (Mn)	12.50
Heavy metals	Lead (Pb)	0.504	Arsenic (As)	0.144
	Mercury (Hg)	UD	Copper (Cu)	5.12
	Cadmium (Cd)	UD	Chromium (Cr)	3.25
Nucleotides	Cytidylic acid	207.44	Uracil nucleotide	1252.29
	Guanine nucleotide	55.31	Hypoxanthine nucleotide	116.61
	Adenine nucleotide	2.84		

UD: undetected.

## Data Availability

Data are contained within the article and supplementary materials.

## Supplementary Materials

The following supporting information can be downloaded at: https://www.mdpi.com/article/10.3390/nu15234906/s1, Figure S1: Flow chart of animal experiment and H&E staining of heart, liver, spleen, and kidney of experimental rats; Figure S2: Mesial and distal bone loss for each group; Figure S3: MetaCyc database-based functional pathway analysis; Figure S4: CR modified the metabolite levels in serum of OP rats; Figure S5: OPLS-DA permutation test; Table S1: Details of antibodies used in IHC; Table S2: The effect of CR on bodyweight and organ indexes in OP rats; Table S3: Relative abundance of top 20 genera; Table S4: The differential metabolites of serum among experimental rats.
