# Supplementary material for: Chroogomphus rutilus Regulates Bone Metabolism to Prevent Periodontal Bone Loss during Orthodontic Tooth Movement in Osteoporotic Rats
Source: Nutrients. 2023 Nov 24;15(23):4906. doi: 10.3390/nu15234906 (PMC10708235; doi:10.3390/nu15234906)
Supplement: Supplementary file 1 [file nutrients-15-04906-s001.zip › nutrients-2681876-supplementary.pdf]

# *Chroogomphus rutilus* Regulates Bone Metabolism to Prevent Periodontal Bone Loss during Orthodontic Tooth Movement in Osteoporotic Rats

**Ying Zhou [1,2], Yanfeng Zhu [3], Xinghui Jin [3], Yongfeng Zhang [4], Jiyu Song [1,2], Zhina Wu [1,2], Yutong Li [1,2], Jingzheng Yi [5], Di Wang [3,4,*] and Min Hu [1,2,*]**

1 Department of Orthodontics, Hospital of Stomatology, Jilin University, Changchun 130021, China; zhouying21@mails.jlu.edu.cn (Y.Z.); songjy20@mails.jlu.edu.cn (J.S.); znwu22@mails.jlu.edu.cn (Z.W.); liyutong@jlu.edu.cn (Y.L.)
2 Jilin Provincial Key Laboratory of Tooth Development and Bone Remodeling, Changchun 130021, China
3 School of Life Sciences, Jilin University, Changchun 130012, China; zhuyf21@mails.jlu.edu.cn (Y.Z.); jinxh21@mails.jlu.edu.cn (X.J.)
4 Engineering Research Center of Chinese Ministry of Education for Edible and Medicinal Fungi, Jilin Agricultural University, Changchun 130118, China; zhangyongfeng@jlau.edu.cn
5 Western Dental Kids, Fresno, CA 93726, USA; yijingzheng1994@gmail.com
* Correspondence: jluwangdi@jlu.edu.cn (D.W.); humin@jlu.edu.cn (M.H.)

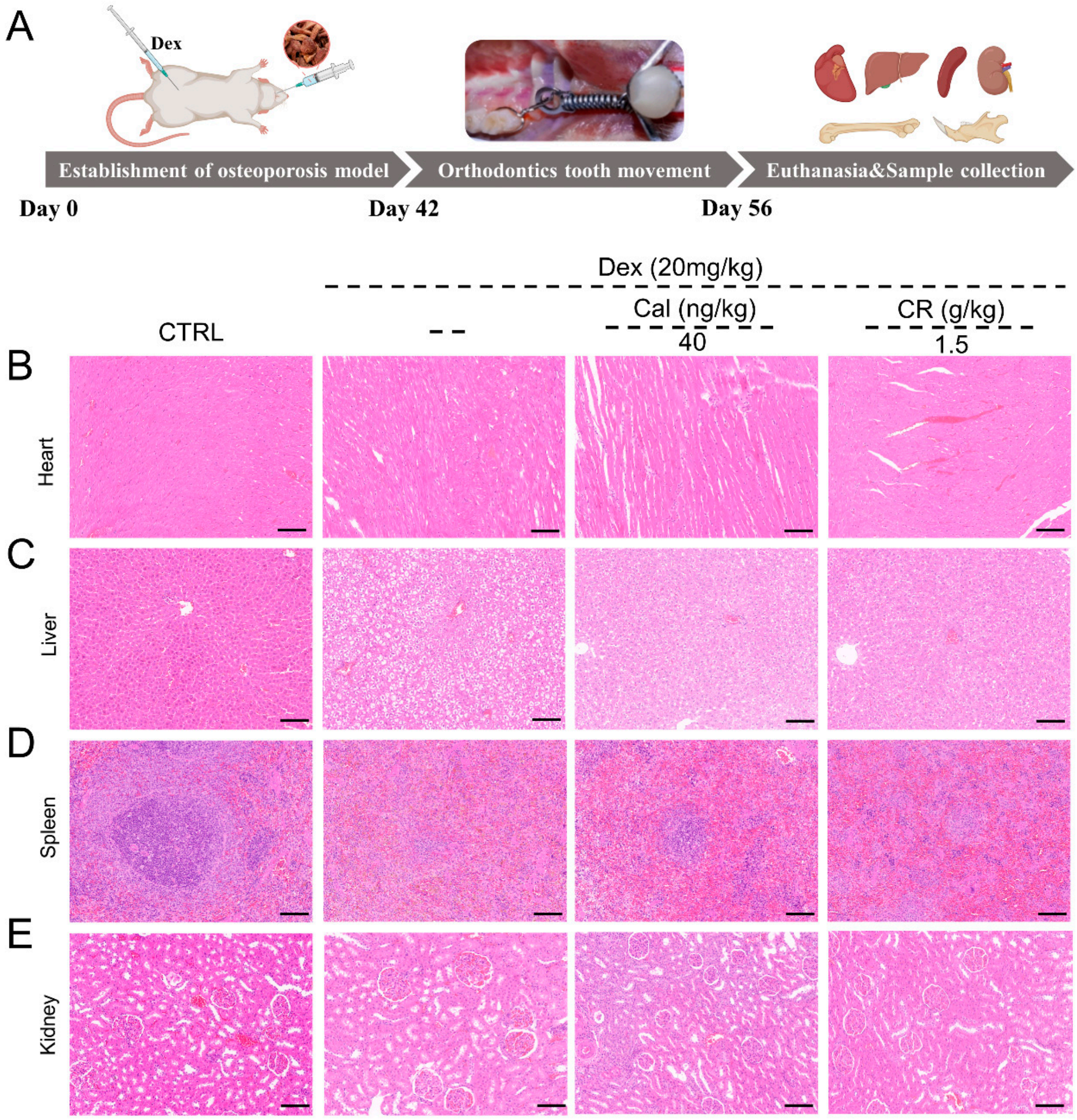


**Figure S1. (A)** Flow chart of animal experiment. H&E staining analysis of the **(B)** heart, **(C)** liver, **(D)** spleen, and **(E)** kidney of experimental rats ($n$ = 3/group, 200×, scale bar: 50 μm

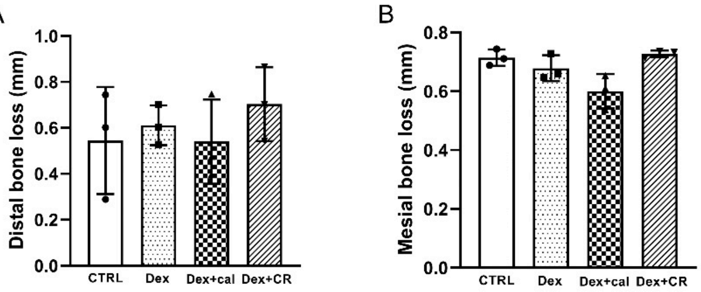


**Figure S2.** Mesial **(A)** and distal **(B)** bone loss for each group ($n$ = 3/group).

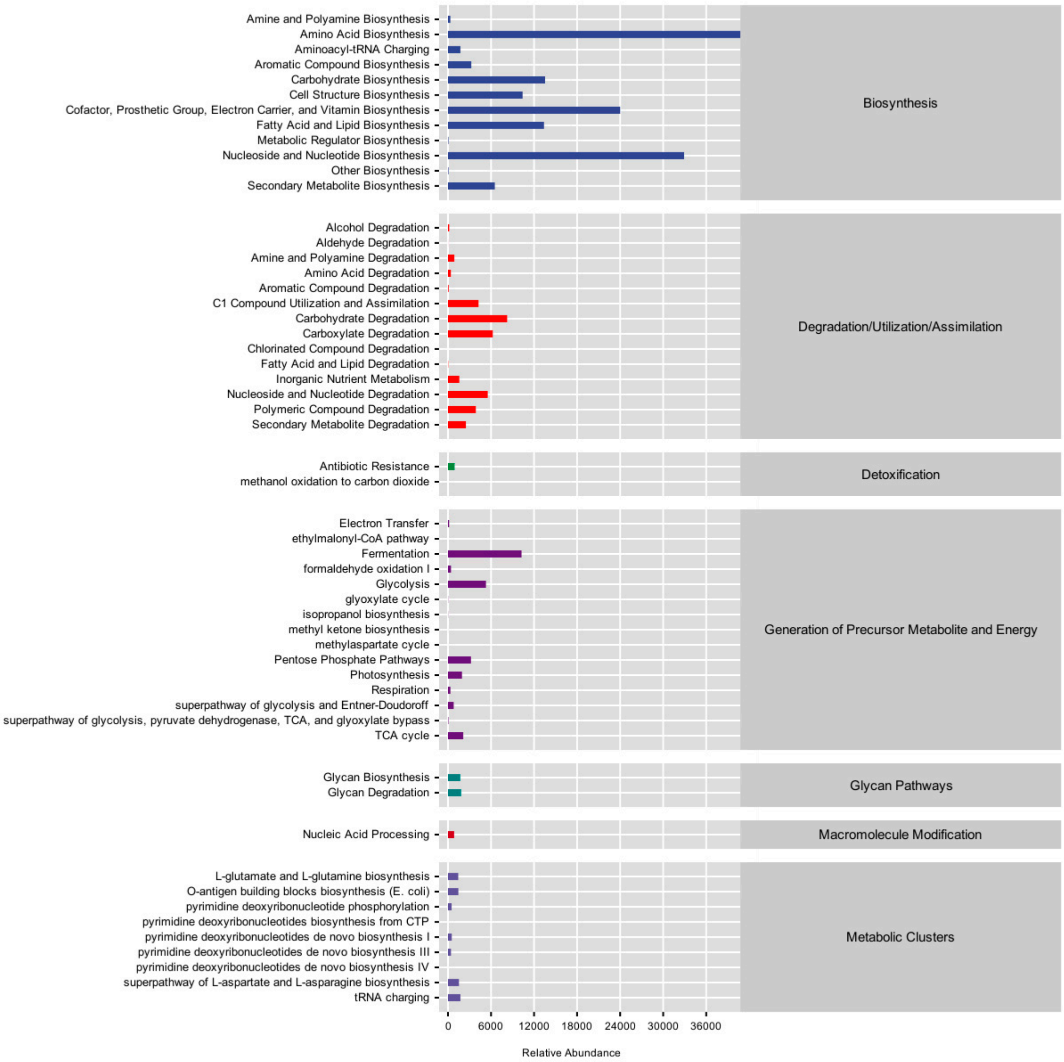


**Figure S3.** MetaCyc database-based functional pathway analysis.

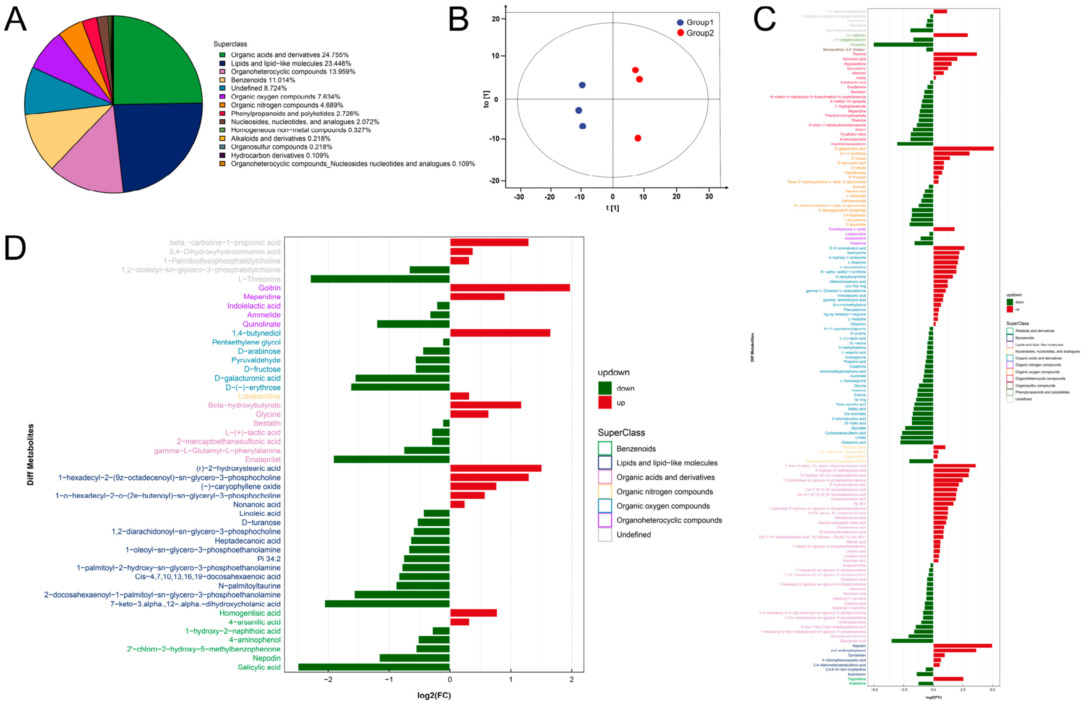


**Figure S4.** CR modified the metabolite levels in serum of osteoporosis (OP) rats. **(A)** Proportion of identified metabolites in each chemical class. **(B)** OPLS-DA score plots for the CTRL and vehicle-treated OP rats. **(C)** Differential fold analysis of significantly different metabolites between CTRL rats and vehicle-treated OP rats. **(D)** Differential fold analysis of significantly different metabolites between vehicle-treated and CR-treated OP rats ($n$ = 3/group).

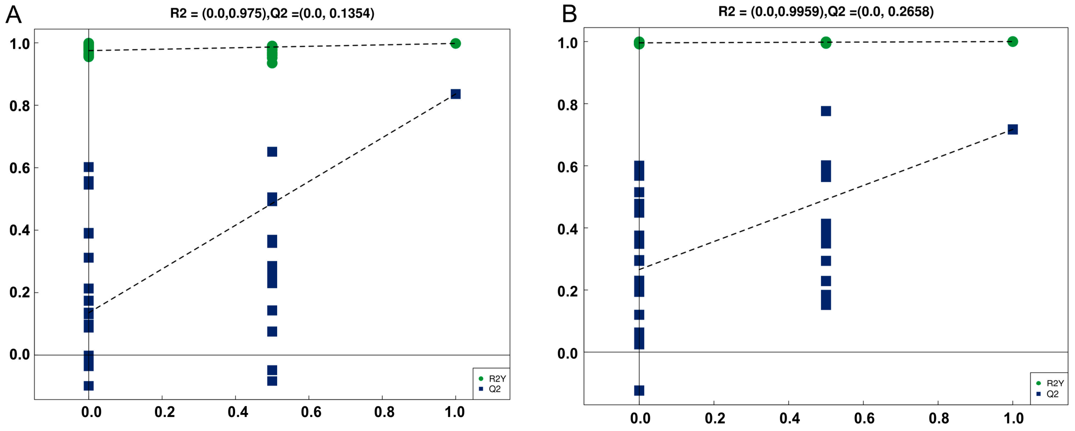


**Figure S5. (A)** OPLS-DA permutation test for the CTRL and vehicle-treated OP rats; **(B)** OPLS-DA permutation test for the vehicle-treated and CR-treated OP rats

**Table S1.** Details of antibodies used in IHC

| Antibody | Molecular | Catalog number | Dilution for IHC | Company | Area |
|---|---|---|---|---|---|
| Runx2 | 57 kDa | AF5186 | 1:200 | Affinity | Jiangsu, China |
| Osterix | 45 kDa | DF7731 | 1:200 | Affinity | Jiangsu, China |
| OPG | 46 kDa | DF6824 | 1:200 | Affinity | Jiangsu, China |
| Rankl | 35 kDa | AF0313 | 1:200 | Affinity | Jiangsu, China |
| Wnt1 | 49 kDa | A2475 | 1:200 | Abclonal | Wuhan, China |
| GSK-3β | 46 kDa | A2081 | 1:200 | Abclonal | Wuhan, China |
| β-catenin | 86 kDa | Bs-1165R | 1:500 | Bioss | Beijing, China |
| TNF-α | 48 kDa | A0277 | 1:200 | Abclonal | Wuhan, China |
| IL-1β | 32 kDa | Bs-0812R | 1:500 | Bioss | Beijing, China |
| IL-6 | 24 kDa | DF6087 | 1:200 | Affinity | Jiangsu, China |

**Table S2.** The effect of CR on bodyweight and organ indexes in OP rats.

| | Days | CTRL | Dex (20 mg/kg) | | |
|---|---|---|---|---|---|
| | | | -- | Cal (40 ng/kg) | CR (1.5 g/kg) |
| Body weight (g) | 0 day | 220.11 ± 11.05 | 224.09 ± 7.84 | 226.53 ± 5.94 | 227.91 ± 7.47 |
| | $7^{th}$ day | 267.30 ± 13.70 | 184.13 ± 9.12$^{###}$ | 191.07 ± 16.63 | 193.36 ± 14.85 |
| | $14^{th}$ day | 323.56 ± 24.16 | 217.66 ± 53.17$^{###}$ | 194.35 ± 18.78 | 193.05 ± 20.67 |
| | $21^{st}$ day | 342.69 ± 31.46 | 216.24 ± 54.80$^{###}$ | 201.98 ± 15.72 | 189.89 ± 25.48 |
| | $28^{th}$ day | 323.59 ± 23.59 | 187.71 ± 7.67$^{###}$ | 196.09 ± 18.30 | 189.18 ± 15.84 |
| | $35^{th}$ day | 354.61 ± 22.33 | 206.91 ± 52.48$^{###}$ | 194.08 ± 15.17 | 189.49 ± 9.44 |
| | $42^{nd}$ day | 361.57 ± 21.07 | 199.44 ± 54.73$^{###}$ | 177.86 ± 22.08 | 209.96 ± 13.58 |
| | $49^{th}$ day | 334.35 ± 19.17 | 199.14 ± 46.87$^{###}$ | 183.62 ± 24.18 | 209.68 ± 19.42 |
| | $56^{th}$ day | 320.51 ± 22.19 | 189.14 ± 44.04$^{###}$ | 193.79 ± 19.47 | 204.58 ± 17.47 |
| Organ Index (%) | Heart | 0.39 ± 0.58 | 0.58 ± 0.78$^{###}$ | 0.51±0.72 | 0.65±1.01 |
| | Liver | 2.45 ± 0.25 | 5.84 ± 0.87$^{###}$ | 3.82±0.53$^{***}$ | 3.81±0.54$^{***}$ |
| | Spleen | 0.14 ± 0.34 | 0.16 ± 0.34 | 0.19±0.42 | 0.16±0.46 |
| | Kidney | 0.68 ± 0.33 | 1.08 ± 0.93$^{###}$ | 1.03±0.89 | 1.05±2.12 |

The data were analyzed using a one-way ANOVA and expressed as mean ± SEM ($n$ = 15/group) $^{###}P < 0.001$ *versus* CTRL rats; $^{***}P < 0.001$ *versus* vehicle-treated OP rats.

**Table S3**. Relative abundance of top 20 genera.

| Taxa | Group1 | Group2 | Group3 |
|---|---|---|---|
| *Faecalibaculum* | 0.1208 | 0.3410 | 0.2241 |
| *Muribaculum* | 0.0552 | 0.0753 | 0.1067 |
| *Lactobacillus* | 0.0064 | 0.1702 | 0.0181 |
| *Akkermansia* | 0.1216 | 0.0000 | 0.0000 |
| *Desulfovibrio* | 0.0156 | 0.0042 | 0.0115 |
| *Bacteroides* | 0.0067 | 0.0032 | 0.0170 |
| *Helicobacter* | 0.0073 | 0.0062 | 0.0120 |
| *Lachnoclostridium* | 0.0071 | 0.0044 | 0.0135 |
| *Ruminococcus* | 0.0115 | 0.0015 | 0.0050 |
| *Escherichia* | 0.0016 | 0.0119 | 0.0028 |
| *Turicibacter* | 0.0092 | 0.0025 | 0.0025 |
| *Prevotella* | 0.0022 | 0.0008 | 0.0092 |
| *Clostridium* | 0.0078 | 0.0034 | 0.0008 |
| *Streptococcus* | 0.0007 | 0.0079 | 0.0012 |
| *Faecalibacterium* | 0.0074 | 0.0000 | 0.0018 |
| *Aerococcus* | 0.0073 | 0.0008 | 0.0000 |
| *Alistipes* | 0.0039 | 0.0010 | 0.0030 |
| *Eisenbergiella* | 0.0010 | 0.0005 | 0.0046 |
| *Oscillibacter* | 0.0026 | 0.0000 | 0.0030 |
| *Flavonifractor* | 0.0030 | 0.0001 | 0.0014 |

Data are presented as the mean ($n$ = 3/group). Group 1: CTRL rats; group 2: vehicle-treated OP rats; group 3: CR-treated OP rats.

**Table S4**. The differential metabolites of serum among experimental rats.

| Metabolites | Group1 | Group2 | Group3 | Group1 *vs* Group2 | | Group2 *vs* Group3 | |
|---|---|---|---|---|---|---|---|
| | | | | VIP | *P*-value | VIP | *P*-value |
| D-(-)-erythrose | 25577805 | 187907396 | 64982362 | 1.579 | 0.020 | 1.677 | 0.031 |
| Linoleic acid | 1190670335 | 1868923221 | 1293795241 | 3.113 | 0.002 | 3.340 | 0.033 |
| D-fructose | 1033144948 | 1597061782 | 1030726929 | 2.837 | 0.018 | 3.390 | 0.027 |
| D-galacturonic acid | 7062557 | 317184933 | 111059612 | 2.215 | 0.013 | 2.100 | 0.048 |
| Pyruvaldehyde | 169621683 | 255993382 | 164406299 | 1.120 | 0.037 | 1.331 | 0.042 |
| (-)-caryophyllene oxide | 15619361 | 9533056 | 15298358 | 1.246 | 0.008 | 1.398 | 0.002 |
| O-LPE | 24063556 | 34451194 | 21924960 | 1.571 | 0.014 | 1.877 | 0.007 |
| Gamma-L-Glutamyl-L-phenylalanine | 9673371 | 23064031 | 13855925 | 1.861 | 0.000 | 1.575 | 0.015 |
| LPE (16:0) | 108250428 | 250992724 | 155883221 | 6.013 | 0.000 | 5.116 | 0.018 |
| C16-20:4 PC | 122796151 | 65626677 | 101577835 | 3.826 | 0.046 | 3.284 | 0.035 |
| PE (16:0/22：6) | 1940802 | 10506550 | 4237365 | 1.467 | 0.001 | 1.311 | 0.037 |
| DOPC | 50772497 | 48838416 | 28472184 | 0.767 | 0.898 | 2.318 | 0.041 |
| Gamma-glutamylvaline | 36913530 | 29485709 | 37748683 | 1.584 | 0.021 | 1.681 | 0.021 |
| Phosphocreatine | 9776492 | 1430439 | 4445177 | 1.475 | 0.001 | 0.907 | 0.042 |
| PE (18:0/22:6) | 1400720 | 9318459 | 4047688 | 1.415 | 0.006 | 1.122 | 0.047 |

Data are presented as the mean ($n$ = 3/group). Differences were considered statistically significant at $P < 0.05$ and VIP > 1. Group 1: CTRL rats; group 2: vehicle-treated OP rats; group 3: CR-treated OP rats.
